# The Effects of Endosomal Toll-like Receptor Inhibitors in an EBV DNA-Exacerbated Inflammatory Bowel Disease Mouse Model

**DOI:** 10.3390/v16040624

**Published:** 2024-04-17

**Authors:** Iman Karout, Zahraa Salhab, Nour Sherri, Elio R. Bitar, Abdul Hamid Borghol, Hady Sabra, Aya Kassem, Omar Osman, Charbel Alam, Sabah Znait, Rayan Assaf, Sukayna Fadlallah, Abdo Jurjus, Jana G. Hashash, Elias A. Rahal

**Affiliations:** 1 Department of Experimental Pathology, Immunology, and Microbiology, American University of Beirut, Beirut 1107-2020, Lebanon; iyk00@mail.aub.edu (I.K.); zs78@aub.edu.lb (Z.S.); nas51@mail.aub.edu (N.S.); erb07@mail.aub.edu (E.R.B.); aib09@mail.aub.edu (A.H.B.); hws06@mail.aub.edu (H.S.); amk123@mail.aub.edu (A.K.); omar.osman221@hotmail.com (O.O.); ca111@aub.edu.lb (C.A.); sz60@aub.edu.lb (S.Z.); ra448@aub.edu.lb (R.A.); smf28@mail.aub.edu (S.F.); 2 Department of Anatomy, Cell Biology and Physiological Sciences, American University of Beirut, Beirut 1107-2020, Lebanon; aj00@aub.edu.lb; 3 Division of Gastroenterology and Hepatology, Mayo Clinic, Jacksonville, FL 32224, USA; alhashash.jana@mayo.edu

**Keywords:** Epstein–Barr virus (EBV), autoimmune diseases, inflammatory bowel disease (IBD), Toll-like receptors (TLR), IL-17A

## Abstract

Epstein–Barr virus (EBV), a *Herpesviridae* family member, is associated with an increased risk of autoimmune disease development in the host. We previously demonstrated that EBV DNA elevates levels of the pro-inflammatory cytokine IL-17A and that inhibiting Toll-like receptor (TLR) 3, 7, or 9 reduces its levels. Moreover, this DNA exacerbated colitis in a mouse model of inflammatory bowel disease (IBD). In the study at hand, we examined whether inhibition of TLR3, 7, or 9 alleviates this exacerbation. Mice were fed 1.5% dextran sulfate sodium (DSS) water and administered EBV DNA. Then, they were treated with a TLR3, 7, or 9 inhibitor or left untreated. We also assessed the additive impact of combined inhibition of all three receptors. Mice that received DSS, EBV DNA, and each inhibitor alone, or a combination of inhibitors, showed significant improvement. They also had a decrease in the numbers of the pathogenic colonic IL-17A+IFN-γ+ foci. Inhibition of all three endosomal TLR receptors offered no additive benefit over administering a single inhibitor. Therefore, inhibition of endosomal TLRs reduces EBV DNA exacerbation of mouse colitis, offering a potential approach for managing IBD patients infected with EBV.

## 1. Introduction

The Epstein–Barr virus (EBV) is a human herpesvirus that causes a variety of immune reactions that could either cause or worsen various EBV-associated disorders [1]. EBV is regarded as a good candidate for initiation or aggravation of autoimmune and inflammatory illnesses because of its capacity to latently infect B cells and elude the immune response [2]. EBV can reactivate which results in EBV DNA shedding [3]. Our group previously reported that giving mice an intra-peritoneal injection of EBV DNA causes their levels of the proinflammatory cytokine interleukin 17A (IL-17A) to rise [4]. Using endosomal Toll-like receptor inhibitors, we reported that this immune signaling pathway was involved in the increase in IL-17A levels [5]. Therefore, EBV induces a pro-autoimmune response causing an elevation in systemic and tissue IL-17A levels, thus acting as a risk factor for the development of autoimmune diseases [6]. Recent studies have suggested that EBV may contribute to the pathophysiology of inflammatory bowel disease (IBD), a chronic inflammatory condition with a rising incidence worldwide [7]. IBD’s etiology is yet unknown; however, it is thought to be multifactorial. Associated factors include genetic predisposition, changes in the composition of the commensal flora in the gut, environmental factors, exposure to antibiotics, and immunodeficiencies [8]. EBV has been found in the intestinal mucosa of IBD patients, and according to various studies up to 60% of IBD patients had intestinal mucosa with EBV-positive cells [9]. This prompted our team to examine the relationship between EBV DNA and IBD aggravation in mouse and *Drosophila melanogaster* models [7,10]. We observed that EBV DNA has a role in aggravating intestinal inflammation in the mouse model. Additionally, we previously demonstrated that inhibiting TLR3, 7, or 9 reduces EBV DNA-enhanced IL-17A levels in mice. Consequently, we aimed in the study at hand to assess whether administering TLR3, 7, or 9 inhibitors alleviates EBV DNA-exacerbated colitis and whether combining these inhibitors has a relevant additive effect on colitis amelioration. We evaluated the disease activity index (DAI), colon length, histological damage, and pro-inflammatory cellular foci in colon tissues in mice. The objective was assessing these receptors as possible therapeutic targets to treat individuals with autoimmune diseases such as IBD that could be aggravated or triggered by an EBV infection or reactivation.

## 2. Materials and Methods

### 2.1. EBV Induction, Extraction, and Quantification

P3HR-1, a Burkitt’s lymphoma cell line, harboring a latent strain of the Epstein–Barr virus (EBV type 2) was used in this study. The cell line was obtained from the American Type Culture Collection (ATCC) (Rockville, MD, USA). It was cultivated in Rosewell Park Memorial Institute (RPMI) 1640 medium (Lonza, Basel, Switzerland) containing 20% fetal bovine serum (Sigma-Aldrich, Darmstadt, Germany) and 1% penicillin–streptomycin (Lonza). The cells were passaged maintaining 70% confluency and were kept at 37 °C with 5% CO_2_. Phorbol 12-myristate 13-acetate (PMA) (Sigma-Aldrich) (Merck KGaA, Darmstadt, Germany) was applied at a concentration of 65 ng/mL to induce EBV. Following induction for 5 days with PMA, cells were subjected to two rounds of centrifugation: one to collect the virus-containing supernatant at 800 rpm for 8 min at room temperature, and the other to pellet the virus at 16,000× *g* for 90 min at 4 degrees. 

To extract DNA, 100 μL of the viral pellet was combined with 100 μL of Tris-HCL saturated phenol, vortexed, and centrifuged at 13,000 rpm for 15 min at 4 degrees. After collecting the upper layer containing the genomic DNA, it was mixed with 100% cold ethanol and 3 M sodium acetate and stored at −80 °C overnight. The DNA pellet was centrifuged, then air-dried, washed with 1 mL of 70% cold ethanol, and then resuspended in 30 μL of nuclease-free water. 

To determine the number of EBV DNA copies, real-time PCR was performed using the Taq Universal SYBER Green Supermix and the Bio-Rad CFX96^TM^ Real Time PCR Detection System (Bio-Rad, Berkeley, CA, USA). Specific primers for the *EBER2* gene were employed for this purpose (Table 1). PCR tubes contained 1 μL of sample DNA in a total volume of 10 μL. The thermal cycling program included a 5 min activation step at 95 °C, followed by 40 cycles of 95 °C and 58 °C for 15 and 30 s, respectively. Using an EBV DNA standard, a curve was generated and used to calculate the number of EBV DNA copies in the P3HR-1 induction preparation. The standard curve was evaluated for acceptability, with a slope of −3.0 to −3.6 and a correlation coefficient of ≥0.98.

### 2.2. Induction of Acute DSS Colitis in C57BL/6J Mice and Treatment Administration

As previously reported by Okayasu et al. [11], C57BL/6J mice were used to induce acute colitis. In this study, C57BL/6J female mice aged 6–8 weeks were employed. The American University of Beirut’s (AUB) Animal Care Facility provided the mice. Protocols were approved by the Institutional Animal Care and Use Committee (IACUC) at AUB.

**Figure 1:**
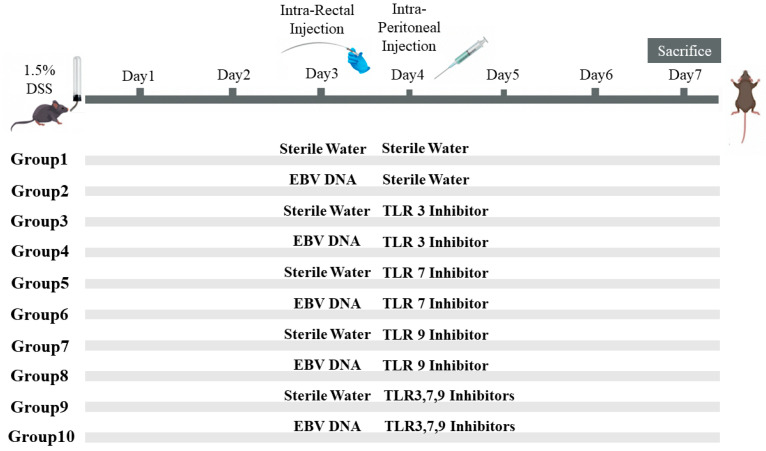
Experimental design used to assess the additive effect of the combination of Toll-like receptor (TLR) 3, 7, and 9 inhibition on the severity of Epstein–Barr virus (EBV) DNA-exacerbated colitis in C57BL/6J mice. All C57BL/6J mice (*n* = 10 per group) received 1.5% dextran sodium sulfate (DSS) in drinking water for 7 days. Group 1 was rectally and intra-peritoneally injected with sterile water. Group 2 was rectally injected with EBV DNA and intra-peritoneally injected with sterile water. Four groups (3, 5, 7, 9) were rectally administered sterile water and intra-peritoneally injected with a TLR3, 7, or 9 inhibitor or a combination of the inhibitors. The other four groups (4, 6, 8, 10) were rectally administered EBV DNA and intra-peritoneally injected with a TLR3, 7, or 9 inhibitor or a combination of the inhibitors.

To assess the additional effect of the combination of Toll-Like Receptor (TLR) Inhibitors 3, 7, and 9 on the severity of colitis in mice injected with EBV DNA, ten groups of 6–8-week-old female mice, with each containing 10 mice, were used (Figure 1). Acute colitis was induced by oral administration of 1.5% (*w/v*) DSS (molecular weight 40 kDa; Chondrex, Redmond, WA, USA) in autoclaved drinking water in all groups of mice. On the third day, Groups 2, 4, 6, 8, and 10 served as experimental groups of mice and each mouse in these groups was administered intra-rectally with 288 × 10^3^ EBV DNA copies in 100 μL of sterile distilled water using a 3.5 French catheter, as a previous study conducted by our group indicated that 288 × 10^3^ EBV DNA copies exacerbated colitis symptoms in the mouse IBD model [7]. The rest of the groups, Groups 1, 3, 5, 7, and 9, served as control mice groups and each mouse received an intra-rectal administration of 100 μL of autoclaved distilled water (the DNA diluent) using a 3.5 French catheter on Day 3. On the fourth day, each mouse in Groups 1 and 2 received intraperitoneal injection of 100 μL of autoclaved distilled water. On the other hand, Groups 3 through 10 served as treatment groups and each mouse received either an intraperitoneal injection of a TLR3 inhibitor (Groups 3 and 4), TLR7 inhibitor (Groups 5 and 6), or TLR9 inhibitor (Groups 7 and 8) as per the doses indicated in Table 2. Groups 9 and 10 received a combination of TLR3, 7, and 9 inhibitors in 400 μL of autoclaved distilled water and the doses indicated in Table 2. The DSS dosage selected relied on optimization studies carried out in order to determine the level of colon damage that allowed for potential further damage brought on by the other treatments. Data from our investigations on systemic EBV and reported EBV levels in colonic tissues were used to determine the EBV DNA dose. The doses of each TLR inhibitor (Humanizing Genomics macrogen) (Geumcheon-gu, Seoul, Republic of Korea) were administered in a specific volume of sterile water as per a previous study performed by our lab [5] (Table 2). 

### 2.3. Mouse Monitoring and Assessment

The severity of colitis in each group of mice was assessed daily starting on Day 0 by recording information about each mouse’s body weight, the consistency of their stools, the presence of occult blood positivity, and the presence of gross blood in the stools. All of these factors went into calculating the DAI as per Cooper et al. [12] (Table 3). On Day 7, all groups were sevoflurane-anesthetized and sacrificed through a midline laparotomy incision immediately followed by heart puncture. The mouse colons were then excised. Each mouse colon’s whole length was measured; a shorter colon is believed to result from more severe colitis [13].

The harvested colon’s lumen was washed with cold phosphate-buffered saline (PBS). A piece of the distal end of the colon closest to the anus was cut off and fixed in 10% formaldehyde overnight followed by embedding in paraffin. Hematoxylin and eosin staining was performed after five micrometer sections from the distal end of the colon were deparaffinized with xylene and rehydrated by passing them through a series of decreasing ethanol washes (100%, 95%, and 70%). After that, the degree of crypt damage and the extent of the inflammation were used to grade the histological damage as described in Table 4 [14]. The cross sections were graded based on severity (inflammatory infiltrates), extent (whether inflammation was limited to the mucosa or whether it had advanced transmurally through the sub-mucosa into the muscles) and damage (basal damage to the crypt/complete crypt loss). After grading these three features, the grades were totaled into one sum, which is the histological damage score. 

### 2.4. Expression Studies

The double-positive count for IL-17A and IFN-γ foci in mouse colon tissues was counted using immunofluorescence labeling on colon sections. The unstained section slides were first heated at 55 °C for 40 min, after which they were deparaffinized by being submerged in xylol twice for ten minutes each time. The sections were then rehydrated by submerging the slides for five minutes in ethanol solutions with decreasing concentrations (100%, 95%, and 75%). The deparaffinization process was followed by submerging the slides twice for 5 min each in distilled water. The slides were then submerged in citrate buffer (pH 6) for 90 min in a water bath set at 60 °C to carry out antigen retrieval. The citrate buffer was prepared from 0.1 M tri-sodium citrate dihydrate and 0.1 M citric acid (18 mL of citric acid, 82 mL of tri-sodium citrate dehydrate, 900 mL distilled water). The slides were taken out from the water bath and allowed to cool in the citrate buffer for 30 min before being washed twice, with each wash involving submerging the slides in distilled water for 5 min. The slides were incubated in the blocking buffer (15% FBS in 1× PBS) for 30 min at room temperature. Then, an antibody dilution buffer was made (1× PBS, 15% FBS, and 0.3% Triton X). The slides were then incubated overnight with fluorochrome-linked primary antibodies. The antibodies used were fluorochrome-labeled Brilliant Violet 605 anti-mouse IL-17A (1:500), Pacific Blue 405 anti-mouse IFN-γ (1:500), and Alexa Fluor 488 anti-mouse FOXP3 (1:500) (Biolegend, San Diego, CA, USA). The slides were then washed three times with 1× PBS, covered with mounting solution (80% glycerol, 223 mM 1,4-diazabicyclo [2.2.2] octane (DABCO), and 4 mM Tris-HCl) and a coverslip before finally being stored at 4 °C. Slides were observed using a Leica DM4B Upright Fluorescence Microscope employing the Leica software. The number of double-positive counts per area was determined using ImageJ v1.54i (National Institutes of Health, Rasband, WS) manually, performed by 2 operators, and expressed as foci per inch^2^.

### 2.5. Statistical Analysis

GraphPad Prism was used to conduct statistical analyses. A normality test, specifically the Shapiro–Wilk test, was utilized to assess whether the data followed a normal distribution. Results indicated that our data did not adhere to a normal distribution, as per the Shapiro–Wilk test. Consequently, the Mann–Whitney U test was used to assess the differences in the colon length, disease activity index scores, histological scoring, and double-positive count for IL-17A and IFN-γ foci in the colon tissues. Outliers were detected using Grubb’s test. *p*-values less than 0.05 were considered statistically significant.

## 3. Results

### 3.1. Administration of Endosomal TLR Inhibitors Ameliorates the Disease Activity Index (DAI) in the EBV DNA-Exacerbated Mouse Model of Acute Colitis

**Figure 2:**
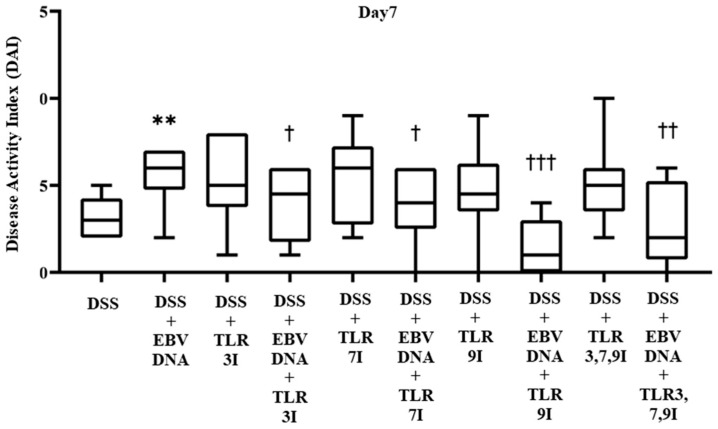
Disease activity index (DAI) scores on Day 7 in control and experimental mouse groups used to assess the additive effect of Toll-like receptors 3,7,9 (TLR3,7,9) inhibition on the severity of Epstein–Barr virus DNA-exacerbated colitis in C57BL/6J mice. The DAI was determined daily by collecting data pertaining to body weight, fecal consistency, and occult blood. ** *p*-value < 0.01, compared to DSS group; † *p*-value < 0.05, compared to DSS + EBV DNA group; †† *p*-value < 0.01, compared to DSS + EBV DNA group; ††† *p*-value < 0.001 compared to DSS + EBV DNA group. TLR3I = TLR3 inhibitor, TLR7I = TLR7 inhibitor, TLR9I = TLR9 inhibitor, TLR3,7,9I = TLR3, 7, and 9 inhibitors.

The effect of TLR3, 7, and 9 inhibition on the DAI in the EBV DNA-exacerbated IBD mouse model was determined on a daily basis by collecting data pertaining to body weight, fecal consistency, and occult blood, and trends were observed to evaluate the severity of colitis. The DAI score showed a gradual increasing trend in all groups administered DSS in their drinking water. By Day 7, the group that received an additional intra-rectal administration of EBV DNA had a significant increase in the DAI score (*p* = 0.0036), with an average DAI score of 5.6, compared to the group that was only administered DSS without the EBV DNA injection, which had an average DAI score of 3.2 (Figure 2). The groups that had received an intra-peritoneal injection of either TLR3 inhibitor, TLR7 inhibitor, or TLR9 inhibitor in addition to the EBV DNA and DSS administration exhibited a decrease in the DAI score, which averaged at 3.8, 3.8, and 1.4, respectively. These DAI scores were significantly lower than that of the group that was only injected with EBV DNA and administered DSS, whose DAI score averaged at 5.6 (*p* = 0.0453, 0.0367, and 0.0002, respectively, for the TLR3, 7, or 9-administered groups). As for the control groups that only received a TLR3 inhibitor, TLR7 inhibitor, or TLR9 inhibitor injection in addition to the DSS, these had an average DAI score of 5.3, 5.3, and 4.7, respectively. The group that had received a combination of TLR3, 7, and 9 inhibitors injection in addition to EBV DNA and DSS administration also exhibited a decrease in the DAI score which averaged 2.6. This DAI score was lower than that of the groups that administered EBV DNA and DSS, injected with TLR3 inhibitor and administered EBV DNA and DSS, and injected with TLR7 inhibitor and administered EBV DNA and DSS. However, this decrease in DAI was only significant when compared the group that was administered with EBV DNA and DSS (*p* = 0.006) (Figure 2). As for the control group that only received a combination of TLR3, 7, and 9 inhibitors and administered DSS, it had an average DAI score of 5. Supplementary information for these results is provided in Table S1.

### 3.2. Administration of Endosomal TLR Inhibitors Increases Colon Lengths in the EBV DNA-Exacerbated Mouse Model of Acute Colitis

**Figure 3:**
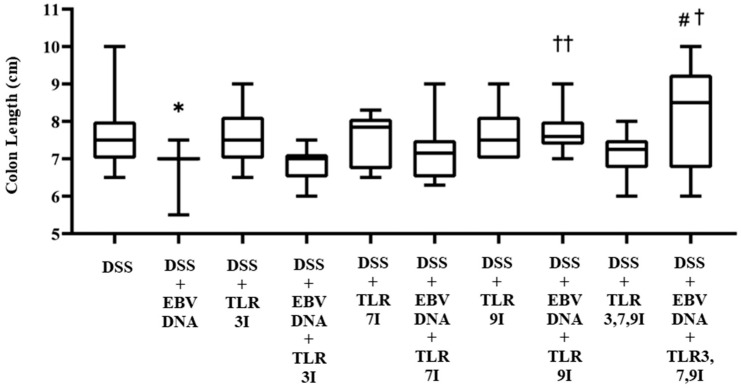
Colon length in control and experimental mouse groups used to assess the additive effect of the combination of Toll-like receptor (TLR) 3, 7, and 9 inhibition on the severity of Epstein–Barr virus (EBV) DNA-exacerbated colitis in C57BL/6J mice. After 7 days, mice were sacrificed and their colon lengths were measured. * *p*-value < 0.05, compared to DSS group; † *p*-value < 0.05, compared to DSS + EBV DNA group; †† *p*-value < 0.01, compared to DSS + EBV DNA group; # *p*-value < 0.05, compared to DSS + EBV DNA + TLR3I group. TLR3I = TLR3 inhibitor, TLR7I = TLR7 inhibitor, TLR9I = TLR9 inhibitor, TLR3,7,9I = TLR3, 7, and 9 inhibitors.

In addition to macroscopically examining colon samples taken from each mouse group on Day 7, which is commonly analyzed in the acute colitis model, the severity of colitis in the IBD mice model was investigated as well by looking for colon length shortening, a marker of inflammation. Consistent with the DAI, the colon length was significantly shorter in the group that received an intra-rectal administration of EBV DNA in addition to the DSS administration (*p* = 0.0190) compared to the colon length of the group that only administered DSS. The latter group had an average colon length of 7.65 cm, whereas that of the former was 6.9 cm (Figure 3). However, the groups that had received an intra-peritoneal injection of either TLR7 inhibitor or TLR9 inhibitor in addition to EBV DNA and DSS administration had an increase in colon length with an average length of 7.21 cm and 7.72 cm, respectively. This increase in colon length was only significant in the group injected with TLR9 inhibitor in addition to EBV DNA and DSS administration compared to the group that was only administered with EBV DNA and DSS (*p* = 0.0021). The group that had received an intra-peritoneal injection of a combination of TLR3, 7, 9 inhibitors in addition to EBV DNA and DSS administration had an increase in colon length compared to the groups that were administered with EBV DNA and DSS, injected with TLR3 inhibitor and administered EBV DNA and DSS, injected with TLR7 inhibitor and administered with EBV DNA and DSS, and injected with TLR9 inhibitor and administered with EBV DNA and DSS with an average length of 8.2 cm. However, this increase in colon length was only significant when compared to the group that was administered EBV DNA and fed DSS (*p* = 0.0346) and to the group that was injected with TLR3 inhibitor and administered EBV DNA and DSS (*p* = 0.0398) (Figure 3). As for the control group that only received a combination of TLR3, 7, and 9 inhibitors by injection in addition to the DSS administration, it had an average colon length of 7.1 cm. Supplementary information for these results is provided in Table S2.

### 3.3. Administration of Endosomal TLR Inhibitors Ameliorates Histological Damage in the EBV DNA-Exacerbated Mouse Model of Acute Colitis

**Figure 4:**
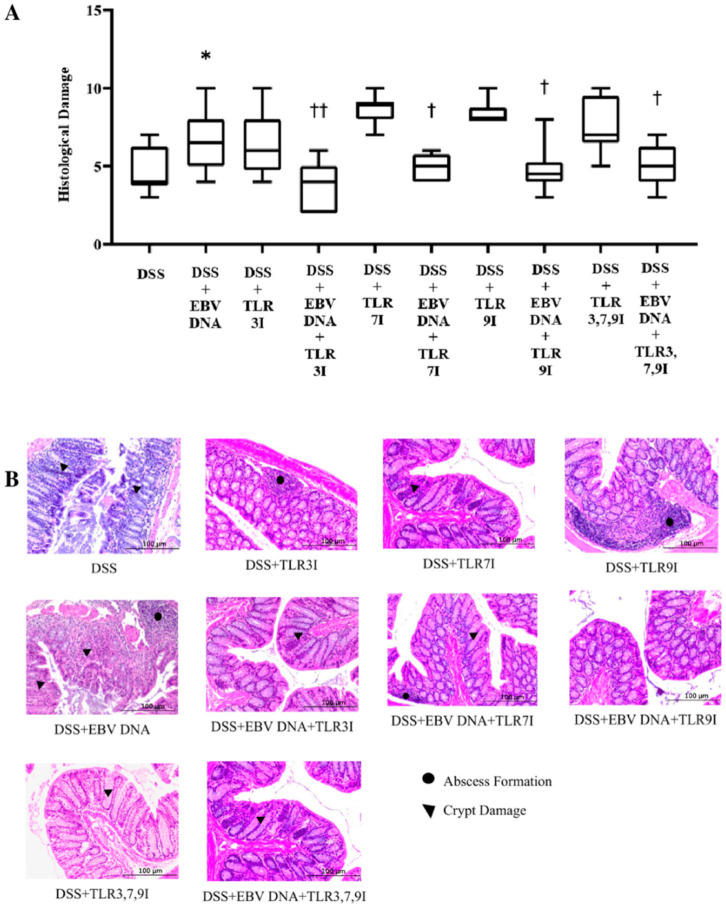
Histological damage scoring in control and experimental mouse groups used to assess the additive effect of the combination of Toll-like receptor (TLR) 3, 7, and 9 inhibition on the severity of Epstein–Barr virus (EBV) DNA-exacerbated colitis in C57BL/6J mice. On Day 7 of the experimental protocol, mice were sacrificed and their histological damage scores were determined. (**A**) Mouse group histology scores. Individual mouse scores, average scores per group, and standard deviations are indicated. (**B**) Hematoxylin and eosin-stained distal sections of mouse colons. * *p*-value < 0.05, compared to DSS group; † *p*-value < 0.05, compared to DSS + EBV DNA group; †† *p*-value < 0.01, compared to DSS + EBV DNA group. TLR3I = TLR3 inhibitor, TLR7I = TLR7 inhibitor, TLR9I = TLR9 inhibitor, TLR3,7,9I = TLR3, 7, and 9 inhibitors.

Histological damage grading was also performed on H&E-stained cross sections of colon samples that were obtained on sacrifice day. Consistent with the DAI and colon shortening, the histological damage score was significantly higher in the group that received an intra-rectal administration of EBV DNA in addition to being fed DSS (*p* = 0.0164) compared to the histological damage score of the group that was only administered DSS (Figure 4). The average histological damage score in the group that received intra-rectal EBV DNA in addition to the DSS administration was 6.7, which was significantly higher than the 4.7 average of the group that was only administered DSS. However, the groups that had received an intra-peritoneal injection of either TLR3 inhibitor, TLR7 inhibitor, or TLR9 inhibitor in addition to EBV DNA and DSS administration had a significant decrease in the histological damage score (*p* = 0.0019, 0.0319, and 0.0195, respectively), with an average histological score of 3.7, 5.2, and 4.8, respectively, compared to the group that received intra-rectal EBV DNA in addition to the DSS administration. Similarly, the group that had received an intra-peritoneal injection of the combination of TLR3, 7, and 9 inhibitors in addition to EBV DNA and DSS administration had a significant decrease in the histological damage score (*p* = 0.0371), with an average score of 5, compared to the group that was only given EBV DNA and administered DSS (Figure 4A). As for the control group that only received the combination of TLR3, 7, and 9 inhibitors in addition to the DSS administration, it had an average histological damage score of 7.6. The average histological score of the group that had received an intra-peritoneal injection of the combination of TLR inhibitors in addition to EBV DNA and DSS administration remained nearly the same as the groups that had received an intraperitoneal injection of either a TLR3, 7, or 9 inhibitor, an intra-rectal administration of EBV DNA, and fed DSS. Figure 4B shows H&E staining of representative colon cross sections in different control and experimental groups. Supplementary information for these results is provided in Table S3.

### 3.4. Administration of Endosomal TLR Inhibitors Decreases the Numbers of Double-Positive IL-17A+/IFN-γ+ Colonic Foci in the EBV DNA-Exacerbated Mouse Model of Acute Colitis

**Figure 5:**
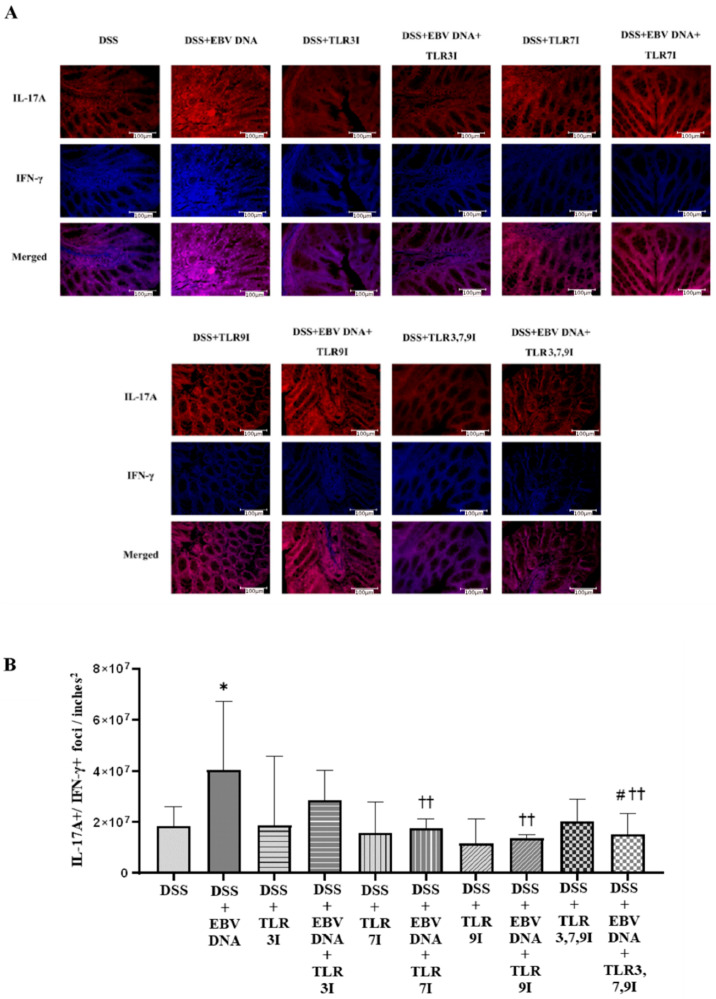
IL-17A+/IFN-γ+ counts in control and experimental mouse groups used to assess the additive effect of the combination of Toll-like receptor (TLR) 3, 7, and 9 inhibition on the severity of Epstein–Barr virus (EBV) DNA-exacerbated colitis in C57BL/6J mice. On Day 7 of the experimental protocol, mice were sacrificed and the colons were collected for immunofluorescence staining for IL-17A and IFN-γ. (**A**) Immunofluorescent staining for IL-17A and IFNY in the colon sections of experimental and control mouse groups. (**B**) IL-17A+/IFN-γ+ double-positive counts in colons of experimental and control mouse groups. * *p*-value < 0.05, compared to DSS group; †† *p*-value < 0.01, compared to DSS + EBV DNA group. # *p*-value < 0.05, compared to DSS + EBV DNA + TLR3I group. TLR3I = TLR3 inhibitor, TLR7I = TLR7 inhibitor, TLR9I = TLR9 inhibitor, TLR3,7,9I = TLR3, 7, and 9 inhibitors.

Cross-sectional samples of the colon taken from the experimental and control groups of mice were assessed for the presence of the pathogenicity-associated IL-17A+/IFN-γ+ colonic foci using immunofluorescence. The number of double-positive IL-17A+/IFN-γ+ counts was the highest in the group that was administered DSS and EBV DNA, the difference in count was significant compared to the group that was only administered DSS (*p* = 0.0111) (Figure 5A). The groups that had received an intra-peritoneal injection of either a TLR3 inhibitor, TLR7 inhibitor, or TLR9 inhibitor in addition to EBV DNA and DSS administration exhibited a further decrease in the number of double-positive IL-17A+/IFN-γ+ counts. The two groups that had received an intra-peritoneal injection of either TLR7 inhibitor or TLR9 inhibitor in addition to the EBV DNA and DSS administration had significantly lower counts than that of the group that was only given EBV DNA and administered DSS (*p* = 0.0023 and 0.0025, respectively). The group that had received a combination of TLR3, 7, and 9 inhibitors in addition to the EBV DNA and DSS also exhibited a decrease in the number of double-positive IL-17A+/IFN-γ+ counts. The number of double-positive IL-17A+/IFN-γ+ counts was lower than that of the groups that were given EBV DNA and DSS, TLR3 inhibitor with EBV DNA and administered DSS, and TLR7 inhibitor with EBV DNA and administered DSS. However, this decrease in the number of double-positive IL-17A+/IFN-γ+ counts was only significant when compared the group that was given EBV DNA and DSS (*p* = 0.0041) and to the group that was injected with TLR3 inhibitor and administered EBV DNA and DSS (*p* = 0.0205) (Figure 5A). Figure 5B shows immunofluorescence staining for IL17-A and IFN-γ in all ten mouse groups.

## 4. Discussion

Epstein–Barr virus (EBV) is a widespread virus that affects over 90% of people worldwide [15]. EBV infections have been linked to various non-malignant conditions like infectious mononucleosis, as well as malignant diseases such as Burkitt lymphoma, Hodgkin lymphoma, and nasopharyngeal carcinoma. EBV is also considered a risk factor for autoimmune and inflammatory diseases, potentially due to its immune-modulating antigens and ability to persist in B cells [2]. There is growing evidence suggesting that EBV infection significantly increases the risk of developing diseases like multiple sclerosis (MS) and rheumatoid arthritis (RA) [4,16].

To investigate the immune-stimulatory properties of EBV and its potential role in triggering autoimmune responses our group examined the potential role of EBV DNA. This immunostimulatory antigen is released during viral reactivation and could contribute to autoimmune disease development or exacerbation. Our findings suggested that administering EBV DNA into mice increased systemic levels of the pro-inflammatory cytokine IL-17A, potentially acting as a risk factor for autoimmune disease [17]. We also examined the roles of TLRs in the response. We hypothesized that EBV DNA activated the TLR9 pathway due to the presence of unmethylated CpG motifs in EBV DNA. Subsequent studies confirmed that EBV DNA activated TLR9 and other TLR family receptors, including TLR3 and TLR7, leading to increased IL-17A production [4,5,17].

We then assessed the association between EBV DNA and disease incidence or exacerbation in human subjects as well as animal models of various inflammatory and autoimmune diseases, including RA and IBD. Studies had suggested that EBV might play a role in the pathogenesis and exacerbation of IBD, with some studies detecting EBV in the intestinal mucosa of IBD patients [18]. Using *Drosophila melanogaster* and mouse models, our group found that EBV DNA had an immunostimulatory role that aggravated gut inflammation, indicating its potential involvement in exacerbating established IBD [7,10].

While excessive production of IL-17A by a microbe-induced Th17 response can promote chronic inflammatory diseases like IBD, it has also been reported that IL-17A plays a protective role in IBD patients. Inhibiting IL-17A with antibodies in a mouse model of DSS-induced IBD worsened gut inflammation, primarily due to weakened intestinal epithelial barrier function, increased intestinal permeability, reduced expression of antimicrobial peptides, and decreased neutrophil aggregation [19,20]. Targeting IL-17A might not only affect inflammatory immune cells but also disrupt homeostatic IL-17A-producing cells, potentially aggravating the condition. On the other hand, cells that produce both IL-17A and IFN-γ are believed to play more of a pathogenic role in inflammatory diseases rather than a homeostatic role [21,22]. Therefore, we aimed to assess whether inhibiting TLR3, 7, or 9 reduces the numbers of colonic IFN-γ+/IL-17+ double-positive foci as well as the intensity of intestinal inflammation in the IBD mouse model administered the EBV DNA. 

In this study, colitis was induced in female C57BL/6 mice aged 6–8 weeks by administering DSS in the drinking water. The DSS-induced gut inflammation model closely mimics human ulcerative colitis (UC) and exhibits symptoms such as weight loss, diarrhea, fecal occult blood, colon shortening, and mucosal ulceration [23,24]. The chosen DSS concentration of 1.5% in drinking water was based on a previous study that induced experimental colitis with moderately severe clinical symptoms and colonic shrinkage [7]. Additionally, our aforementioned previous studies had shown that intra-rectally administered 288 × 10^3^ EBV DNA copies exacerbated colitis symptoms in the mouse IBD model [7]. Therefore, this EBV DNA concentration was also used in the current study. Furthermore, we previously demonstrated that intra-peritoneal injection of 57.5 µg of IRS661 (TLR7 inhibitor), 250 µg of ODN2006 (TLR3 inhibitor), and 56 µg of ODN2088 (TLR9 inhibitor), in 100 µL, 200 µL, and 100 µL of sterile water, respectively, reduced systemic IL-17A levels in response to EBV DNA in mice. Consequently, the same doses were employed in the present study.

The additive/synergistic effect of the combination of TLR3, 7, and 9 inhibition on EBV DNA-exacerbated colitis was assessed based on trends in the DAI, macroscopic assessments of the colon, histological damage grading of H&E-stained colon cross sections, and immunofluorescence staining of pro-inflammatory markers in colon cross sections.

In line with findings from a prior study conducted by our research team [25], the group that received an intra-rectal injection of EBV DNA alongside DSS administration exhibited a notably higher DAI score by Day 7 compared to the group that solely received DSS. This suggests that EBV DNA exacerbates the clinical symptoms of colitis in the mouse model of the disease. Conversely, the group that received an intra-peritoneal injection of the combination of TLR3, 7, and 9 inhibitors in addition to EBV DNA and DSS administration displayed a significantly lower DAI score by Day 7 compared to the group that received an intra-rectal administration of EBV DNA alongside DSS but an insignificant decrease in the DAI when compared to the groups that received an intra-rectal administration of EBV DNA alongside DSS and an intra-peritoneal injection of TLR3, 7, or 9 inhibitor. This indicates that the combination of TLR3, 7, and 9 inhibition mitigates the severity of the clinical course. However, no additive or synergistic effect was observed.

Consistent with the DAI scores, the group that received an intra-rectal administration of EBV DNA alongside DSS exhibited shorter colon lengths by Day 7 compared to the group that only received DSS. Conversely, the group that received an intra-peritoneal injection of the combination of TLR3, 7, and 9 inhibitors in addition to EBV DNA and DSS administration had a significantly longer colon length compared to the group that received an intra-rectal administration of EBV DNA alongside DSS and to the group that received an intra-rectal administration of EBV DNA alongside DSS and intra-peritoneal injection of TLR3 inhibitor. Similarly, the group that received an intra-peritoneal injection of the combination of TLR3, 7, and 9 inhibitors in addition to EBV DNA and DSS administration had a longer colon length compared to the groups that received an intra-rectal administration of EBV DNA alongside DSS and an intra-peritoneal injection of TLR7 or 9 inhibitors. This suggests that the combination of TLR3, 7, and 9 inhibition contributed to further elongation of the colon by reducing the severity of inflammation. However, this elongation was significant only when compared to TLR3 inhibition. 

In accordance with the DAI results, the group that received an intra-rectal administration of EBV DNA alongside DSS exhibited a significantly higher histological damage score by Day 7 compared to the group that only received DSS. This suggests that EBV DNA exacerbates the clinical symptoms of colitis in the mouse model of the disease. In alignment with both the DAI index score and colon length measurements, the group that received an intra-peritoneal injection of the combination of TLR3, 7, and 9 inhibitors in addition to EBV DNA and DSS administration displayed a significantly lower histological damage score by Day 7 compared to the group that had received an intra-rectal administration of EBV DNA alongside DSS. However, this group’s histological damage score remained nearly the same compared to the groups that had received an intra-rectal administration of EBV DNA alongside DSS and an intra-peritoneal injection of TLR3, 7, or 9 inhibitors. This indicates that the combination of TLR3, 7, and 9 inhibition mitigated the severity of the clinical course but did not have an additive or synergistic effect when compared to TLR3, 7, or 9 inhibition alone. The groups administered DSS and TLR 3, 7, or 9 inhibitors had a higher histological damage score than the group administered DSS only. On the other hand, when given to mice, a TLR9 antagonist (ODN2088) increased IL-17A levels. That did not, however, have a substantial impact on the levels of IL-17A when mouse PBMCs were used in isolation. ODN2088 was similarly seen to raise human CD4 T cell IL-17A levels [26]. This implies that even if ODN2088 inhibits TLR9 and the MyD88 pathway, it may activate other mediators. These results could be explained by the intricate interactions between pattern recognition receptors (PRRs). In addition to endosomal TLRs, nucleic acids are detected in the cytosol by a number of receptors that converge at the STING protein, including AIM2, IFI16, cGAS, and DLM-1/ZBP1 [27,28]. Phosphorylation of IRF3 and IRF7 by TBK1 triggers proinflammatory responses in response to STING activation. It is hence possible that the inhibitors used in our study could inhibit a TLR but activate other inflammatory mediators. The diverse reactions to TLR inhibitors demonstrate the intricacy of the cell types and nucleic acid-responsive pathways involved. Nevertheless, endosomal TLRs are crucial to the response to EBV DNA, as evidenced by the steady drop in IL-17A levels observed in response to EBV DNA when TLR inhibitors are used [5].

IL-17A plays a crucial role in the immune response involving Th17 cells, which recruit and activate granulocytes. Th17 cells are also implicated in intestinal inflammation, infiltrating the gastrointestinal mucosa of Crohn’s disease and ulcerative colitis patients, leading to excessive IL-17A release [29]. While the Th17 response is essential for clearing certain pathogens by recruiting neutrophils to inflammation sites, it can contribute to chronic inflammatory diseases like IBD when microbial agents trigger uncontrolled IL-17A production. Additionally, studies in CD45RB models suggest a connection between colitis development and elevated IFN-γ levels mediated by the Th1 response, further supporting the inflammatory role of IFN-γ in IBD [30]. An interplay between IL-17A and IFN-γ that regulates gut inflammation has been observed. IL-17A+ IFN-γ+ double-positive CD4+ T cells were detected in the inflamed mucosa of IBD patients, contributing to elevated IFN-γ and IL-17A levels in the gut compared to healthy individuals [21]. These double-positive cells have distinct properties and are more cytotoxic and potent than Th1 lineage cells [21,22]. 

When staining for these two markers, IL-17A and IFN-γ, the group that had received an intra-rectal administration of EBV DNA in addition to DSS had a significant increase in IL-17A+/IFN-γ+ count compared to the group that was only administered DSS. Regarding the group that were administered DSS, EBV DNA, and the combination of TLR3, 7, and 9 inhibitors, the number of double-positive IL-17A+/IFN-γ+ foci was lower than that of the groups that were administered with EBV DNA and DSS, injected with TLR3 inhibitor and administered with EBV DNA and DSS, and injected with TLR7 inhibitor and administered with EBV DNA and DSS. However, this decrease in the number of double-positive IL-17A+/IFN-γ+ counts was only significant when compared the group that was administered with EBV DNA and DSS and to the group that was injected with TLR3 inhibitor and administered EBV DNA and DSS (*p* = 0.0205). This result aligns with the (DAI) result observed above whereby the group that was administered DSS, EBV DNA and the combination of TLR3, 7, and 9 inhibitors had a lower DAI than that of the groups that were administered EBV DNA and DSS, injected with TLR3 inhibitor and administered with EBV DNA and DSS, or injected with TLR7 inhibitor and administered with EBV DNA and DSS. Therefore, this decrease in the DAI observed in the group administered DSS and EBV DNA and the combination of TLR inhibitors may be due to the decrease in the double-positive IL-17A+/IFN-γ+ counts. 

In summary, our research demonstrates that inhibiting TLR3, 7, and 9 in combination has mitigated the extent of intestinal inflammation exacerbated by EBV DNA in a mouse model of IBD. TLR9 inhibitor is the one appearing to be the most effective in inhibiting the effect of EBV DNA injection in IBD mouse model. However, there is no added benefit to combining the inhibitors over administering either of them. It is worthy to note that these inhibitors may inhibit a TLR but may stimulate another mediator resulting in inflammatory responses. Future investigations could explore the underlying pathway that requires stimulation of all three endosomal TLRs to induce this pathology. The ultimate objective would be performing clinical trials on IBD patients infected with EBV. These receptors might serve as possible therapeutic targets to treat individuals with autoimmune diseases such as IBD that could be aggravated or triggered by an EBV infection.

## Figures and Tables

**Table 1:** Primers and annealing temperatures used for real time PCR.

Gene	Primers	Annealing Temperature
*EBER2*	F: 5′-CCCTAGTGGTTTCGGACACA-3′	58 °C
	R: 5′-ACTTGCAAATGCTCTAGGCG-3′	58 °C

**Table 2:** Toll-like receptors (TLR) specifications and doses.

TLR Inhibitor Specification	Diluent	Route of Administration	Dose per Volume (μL)
ODN 2088 (TLR9 inhibitor)	Water	Intraperitoneal	56 μg in 100 μL
ODN2006 (TLR3 inhibitor)	Water	Intraperitoneal	250 μg in 200 μL
IRS661 (TLR7 Inhibitor)	Water	Intraperitoneal	57.5 μg in 100 μL

**Table 3:** Disease activity index (DAI) scoring system used for evaluation of dextran sodium sulfate (DSS) colitis in C57BL/6J mice.

	Clinical Parameters		
Score	Weight Loss (%)	Stool Consistency	Blood in Feces
0	None	Normal	None
1	1–5	-	-
2	6–10	Loose Stool	Occult Bleeding
3	11–15	-	-
4	>15	Diarrhea	Gross Bleeding

**Table 4:** Histological damage grading system used for evaluation of dextran sodium sulfate (DSS) colitis in C57BL/6J mice. The score is a composite of scoring: severity, extent, and crypt damage. Added scores range between 0 and 10.

Featured Score	Grade	Description
Inflammation Severity	0	None
	1	Mild
	2	Moderate
	3	Severe
Inflammation Extent	0	None
	1	Mucosa
	2	Submucosa
	3	Transmural
Crypt Damage	0	None
	1	1/3 Basal Damage
	2	2/3 Basal Damage
	3	Crypt loss with present surface epithelium
	4	Crypt loss and surface epithelium loss

## Data Availability

High-resolution images included in Figure 4 and Figure 5 can be downloaded, respectively, from URL (accessed on 15 April 2024) https://drive.google.com/drive/folders/1goR7d3b9gPJgN3QPx4O63L-_u5VfpBpp?usp=sharing and URL (accessed on 15 April 2024) https://drive.google.com/drive/folders/1Sz_C7bDEamYdMc3ry3n8H5XGlC2Hii2o?usp=sharing. Other data are available upon request submitted to the corresponding author.

## Supplementary Materials

The following supporting information can be downloaded at: https://www.mdpi.com/article/10.3390/v16040624/s1, Table S1: Disease Activity Index (DAI); Table S2: Colon Length; Table S3: Histological Damage.
