# Supplementary material for: The Effects of Endosomal Toll-like Receptor Inhibitors in an EBV DNA-Exacerbated Inflammatory Bowel Disease Mouse Model
Source: Viruses. 2024 Apr 17;16(4):624. doi: 10.3390/v16040624 (PMC11054613; doi:10.3390/v16040624)
Supplement: Supplementary file 1 [file viruses-16-00624-s001.zip › viruses-2913723-supplementary.pdf]

Table S1. Disease Activity Index (DAI)

Group	Mouse	DAI (Day 7)
DSS	A1	3
	A2	2
	A3	3
	A4	3
	A5	5
	B1	4
	B2	2
	B3	2
	B4	3
	B5	5
DSS+EBV DNA	856	6
	A2	7
	A3	5
	A4	7
	A5	7
	B1	2
	B2	7
	B3	6
	B4	4
	B5	5
DSS+TLR3 Inhibitor	A1	4
	A2	1
	A3	3
	A4	8
	A5	6
	B1	8
	B2	7
	B3	8
	B4	4
	B5	4
DSS+EBV DNA+TLR3 Inhibitor	A1	4
	A2	6
	A3	2
	A4	5
	A5	2
	B1	5
	B2	6
	B3	1
	B4	6
	B5	1
DSS+TLR7 Inhibitor	A1	8
	A2	7
	A3	7

	A4	3
	A5	6
	B1	3
	B2	2
	B3	9
	B4	2
	B5	6
**DSS+EBV DNA+TLR7 Inhibitor**	A1	6
	A2	6
	A3	6
	A4	4
	A5	0
	B1	3
	B2	1
	B3	3
	B4	4
	B5	5
**DSS+TLR9 Inhibitor**	A1	6
	A2	0
	A3	6
	A4	7
	A5	4
	B1	9
	B2	2
	B3	4
	B4	4
	B5	5
**DSS+EBV DNA+TLR9 Inhibitor**	A1	0
	A2	2
	A3	0
	A4	2
	A5	0
	B1	4
	B2	3
	B3	0
	B4	3
	B5	0
**DSS+TLR3,7,9 Inhibitors**	1	4
	2	6
	3	5
	4	2
	5	6
	850	10
	849	6
	878	5
	847	4

	846	2
**DSS+EBV DNA+TLR3,7,9 Inhibitors**	1	1
	2	1
	3	0
	4	3
	5	2
	876	6
	844	5
	843	6
	842	2
	874	0

Table S2. Colon Length

Group	Mouse	Colon Length (cm)
DSS	A1	7
	A2	7.5
	A3	7
	A4	10
	A5	6.5
	B1	7.5
	B2	7.5
	B3	8
	B4	8
	B5	7.5
DSS+EBV DNA	856	5.5
	A2	7.5
	A3	7
	A4	7
	A5	7
	B1	6.8
	B2	7
	B3	7.2
	B4	7
	B5	7
DSS+TLR3 Inhibitor	A1	9
	A2	6.5
	A3	8
	A4	7
	A5	7
	B1	7
	B2	8
	B3	8.5
	B4	7
	B5	8
DSS+EBV DNA+TLR3 Inhibitor	A1	6.5
	A2	6
	A3	7
	A4	7
	A5	7
	B1	7.5
	B2	6.5
	B3	7
	B4	6.5
	B5	7.5
DSS+TLR7 Inhibitor	A1	7.7
	A2	8
	A3	7.2

	A4	6.5
	A5	8
	B1	8.3
	B2	6.8
	B3	6.5
	B4	8.3
	B5	8
**DSS+EBV DNA+TLR7 Inhibitor**	A1	7.3
	A2	7
	A3	7.5
	A4	6.5
	A5	7.5
	B1	7
	B2	7.5
	B3	9
	B4	6.5
	B5	6.3
**DSS+TLR9 Inhibitor**	A1	7.5
	A2	7.5
	A3	7
	A4	7
	A5	7
	B1	9
	B2	8
	B3	7.5
	B4	7
	B5	8.5
**DSS+EBV DNA+TLR9 Inhibitor**	A1	7
	A2	8
	A3	8
	A4	9
	A5	7.5
	B1	7
	B2	8
	B3	7.7
	B4	7.5
	B5	7.5
**DSS+TLR3,7,9 Inhibitors**	1	7
	2	7.5
	3	7.5
	4	8
	5	7
	850	7.5
	849	7
	878	7.5
	847	6

	846	6
**DSS+EBV DNA+TLR3,7,9 Inhibitors**	1	10
	2	10
	3	8
	4	9
	5	9
	876	8
	844	6
	843	7
	842	6
	874	9

Table S3. Histological Damage

Group	Mouse	Histological Damage
DSS	A1	4
	A2	6
	A3	5
	A4	4
	A5	3
	B1	4
	B2	7
	B3	7
	B4	4
	B5	3
DSS+EBV DNA	856	10
	A2	8
	A3	5
	A4	4
	A5	5
	B1	6
	B2	8
	B3	6
	B4	7
	B5	8
DSS+TLR3 Inhibitor	A1	8
	A2	8
	A3	5
	A4	10
	A5	6
	B1	4
	B2	4
	B3	6
	B4	6
	B5	6
DSS+EBV DNA+TLR3 Inhibitor	A1	2
	A2	2
	A3	5
	A4	2
	A5	2
	B1	5
	B2	5
	B3	5
	B4	6
	B5	3
DSS+TLR7 Inhibitor	A1	9
	A2	8
	A3	9

	A4	7
	A5	8
	B1	10
	B2	9
	B3	8
	B4	9
	B5	9
**DSS+EBV DNA+TLR7 Inhibitor**	A1	5
	A2	6
	A4	6
	A5	4
	B1	5
	B2	5
	B4	4
	B5	4
**DSS+TLR9 Inhibitor**	A1	8
	A3	8
	A4	10
	B1	9
	B2	8
	B3	8
	B4	8
	B5	8
**DSS+EBV DNA+TLR9 Inhibitor**	A1	6
	A2	5
	A3	4
	A4	3
	A5	5
	B1	4
	B2	4
	B3	8
	B4	5
	B5	4
**DSS+TLR3,7,9 Inhibitors**	1	7
	2	9
	3	7
	4	7
	850	8
	849	10
	878	6
	847	5
	846	10
**DSS+EBV DNA+TLR3,7,9 Inhibitors**	1	3
	2	4
	3	4

	4	4
	5	5
	876	5
	844	6
	843	7
	842	7
	874	5
